# 2417. Epidemiology of Central Line-Associated Bloodstream Infections Present on Hospital Admission: A Multicenter Study

**DOI:** 10.1093/ofid/ofad500.2037

**Published:** 2023-11-27

**Authors:** Patrick R Ching, Opeyemi Oladapo-Shittu, Yea-Jen Hsu, Heather Saunders, Alejandra B Salinas, Taylor N Helsel, Stephanie Mayoryk, Avinash Gadala, Sara E Cosgrove, Clare Rock, Eili Klein, Lisa Maragakis, Lisa Pineles, Anthony Harris, Carlos Mejia-Chew, Sara C Keller

**Affiliations:** Division of Infectious Diseases, Department of Medicine, Washington University School of Medicine, Richmond, Virginia; Johns Hopkins University, Baltimore, Maryland; Johns Hopkins Bloomberg School of Public Health, Baltimore, Maryland; Johns Hopkins University, Baltimore, Maryland; Johns Hopkins University School of Medicine, Baltimore, Maryland; Johns Hopkins University School of Medicine, Baltimore, Maryland; University of Maryland School of Medicine, Shrewsbury, Pennsylvania; Johns Hopkins Health System, Baltimore, Maryland; Johns Hopkins School of Medicine, Baltimore, MD; Johns Hopkins School of Medicine, Baltimore, MD; Johns Hopkins School of Medicine, Baltimore, MD; Johns Hopkins Medicine, Baltimore, MD; University of Maryland School of Medicine, Shrewsbury, Pennsylvania; University of Maryland School of Medicine, Shrewsbury, Pennsylvania; Washington University in St Louis, St. Louis, Missouri; Johns Hopkins University School of Medicine, Baltimore, Maryland

## Abstract

**Background:**

National estimates of central line-associated bloodstream infections (CLABSI) focus on acute care hospital settings. Little is known about CLABSI that develop outside of hospitals and are present on admission (CLABSI-POA). We aimed to describe the epidemiology of CLABSI-POA to develop appropriate prevention strategies.

**Methods:**

We performed a retrospective cohort study of all adult and pediatric patients admitted from 11/01/2020 to 10/31/2021 in two large medical systems in the US, one in Maryland and one in Missouri. Adapting the National Healthcare Safety Network acute care CLABSI definition, we included patients who had a central venous catheter (CVC) present on admission (or had a CVC discontinued within 72 hours prior to admission) and a positive blood culture within 72 hours before and/or after admission. We performed descriptive statistics to characterize the epidemiology of CLABSI-POA.

**Results:**

Of 402 patients with CLABSI-POA, 52% were male, 68% were age ≥45 years, and 45% had Medicare insurance as primary payer. Half of the cases had a hospitalization 30 days prior to the index admission, 22% had at least one prior CLABSI, and 40% received chemotherapy in the last 6 months. The most common indications for catheter placement were chemotherapy (34%), total parenteral nutrition (21%), dialysis (15%), and outpatient parenteral antimicrobial therapy (11%). Attribution of CLABSI-POA based on site where the catheter was routinely accessed included oncology clinics (34%), home infusion therapy (32%), hemodialysis centers (12%), and skilled nursing facilities (9%). The most common causative microorganisms were coagulase-negative staphylococci (23%), *Staphylococcus aureus* (21%), *Enterococcus* spp. (12%), *Candida* spp. (11%), *Escherichia coli* and *Klebsiella pneumoniae* (10% each), and *Pseudomonas aeruginosa* (7%). Forty-five (11%) cases were due to multidrug resistant organisms. Overall in-hospital mortality was 10%.

**Figure:**
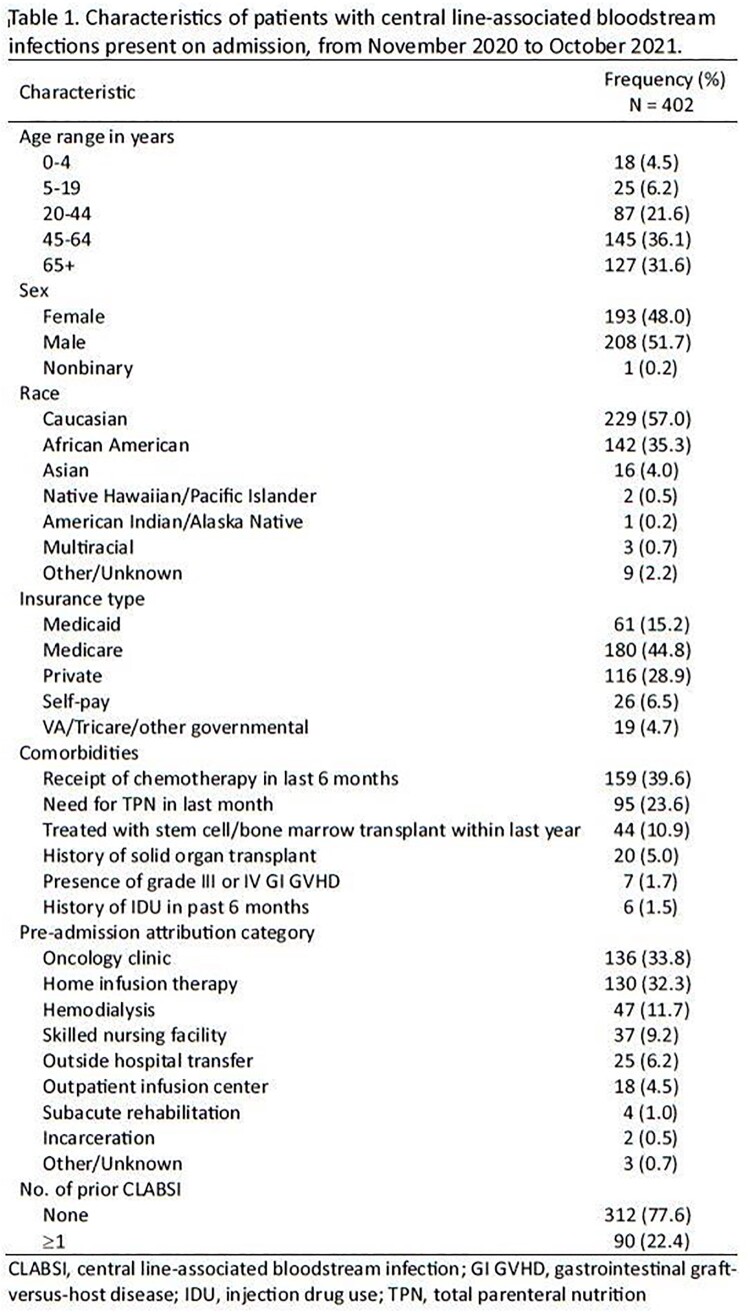

**Conclusion:**

CLABSI-POA were common in those attending oncology clinics and receiving home infusion therapy. Lack of routine surveillance of CLABSI-POAs leads to an underestimation of the burden of CLABSIs in the healthcare system. Tailored prevention strategies should be focused in those groups at higher risk.

**Disclosures:**

**Stephanie Mayoryk, MAS BSN RN CIC**, PDI: Honoraria **Sara E. Cosgrove, MD, MS**, Debiopharm: Advisor/Consultant|Duke Clinical Research Institute: Advisor/Consultant **Carlos Mejia-Chew, MD**, INSMED: Grant/Research Support|RevImmune: Grant/Research Support **Sara C. Keller, MD, MPH, MSPH**, Pfizer: Advisor/Consultant

